# Validation of the individualised neuromuscular quality of life for the USA with comparison of the impact of muscle disease on those living in USA versus UK

**DOI:** 10.1186/1477-7525-9-114

**Published:** 2011-12-16

**Authors:** Reza Sadjadi, Kelly A Vincent, Alison J Carr, Jessica Walburn, Victoria L Brooks, Shree Pandya, John T Kissel, Carlayne E Jackson, Michael R Rose

**Affiliations:** 1 Department of Neurology, King's College Hospital, Denmark Hill, London, SE5 9RS, UK; 2 Department of Rheumatology, King's College Hospital, Denmark Hill, London, SE5 9RS, UK; 3 Department Of Neurology University of Rochester Medical Center, Box 673, 601 Elmwood Avenue, Rochester, New York, NY 14642, USA; 4 Division of Neuromuscular Medicine, The Ohio State University, 395 W. 12th AvenueColumbus, Ohio, OH 43210, USA; 5 Department of Neurology, University of Texas Health Science Center, 7703 Floyd Curl Drive, San Antonio, Texas, TX 78229, USA

**Keywords:** Adult muscle disease, Quality of life

## Abstract

**Background:**

The Individualised Neuromuscular Quality of Life (INQoL) questionnaire is a published muscle disease specific measure of QoL that has been validated using both qualitative and quantitative methods in a United Kingdom population of adults with muscle disease. If INQoL is to be used in other countries it needs to be linguistically and culturally validated for those countries. It may be important to understand any cultural differences in how patients rate their QoL when applying QoL measures in multi-national clinical trials.

**Methods:**

We conducted a postal survey of QoL issues in US adults with muscle disease using an agreed translation, from UK to US English, of the same questionnaire as was used in the original construction of INQoL. This questionnaire included an opportunity for free text comments on any aspects of QoL that might not have been covered by the questionnaire. We examined the responses using both quantitative and qualitative approaches. The frequency of the responses in US versus UK populations was compared using appropriate correlation tests and Rasch analysis. A phenomenological approach was used to guide the qualitative analysis and facilitate the exploration of patients' perceptions and experiences.

**Results:**

The US survey received 333 responses which were compared with 251 UK survey responses.

We found that INQoL domains covered all the issues raised by US subjects with no additional domains required. The experiences of those with muscle disease were remarkably similar in the US and UK but there were differences related to the impact of muscle disease on relationships and on employment which was greater for those living in the United States. The greater impact on employment was associated with a higher importance rating given to employment in the US. This may reflect the lower level of financial support for those who are unemployed, and the loss of employment related health benefits.

**Conclusions:**

INQoL is appropriate for use in US population but there may be differences in the importance that US subject attach to certain aspects of QoL that could be the basis for further study.

If these differences are confirmed then this may have implications for the interpretation of QoL outcomes in multi-national trials.

## Introduction

Muscle diseases (MD) are a group of conditions that can be acquired or genetic and which result in progressive shrinking and weakness of the skeletal muscle such as to cause varying degrees of disability. The individual muscle diseases differ in their age of onset, their rate of progression and their pattern of weakness which in turn dictates the nature and extent of the disability that they cause. The disability caused by MD impacts upon quality of life. The Individualised Neuromuscular Quality of Life (INQoL) questionnaire is a MD specific measure of QoL that has been validated using a UK population [1]. The construction of INQoL was based upon both qualitative and quantitative methods which established the face, content and construct validity, the reliability (test re-test) and to some extent the responsiveness of INQoL. Because QoL is a patient reported subjective measure it is important to ensure that it remains valid when used in countries other than the UK where language and culture vary. In order to achieve this linguistic and cultural validity for the use of INQoL for MD patients in the United States, we needed to first agree upon an American English translation. We also needed to ensure that the QoL domains identified in the UK research were also appropriate for patients from the United States and check that there were no additional domains that might require inclusion. Most publications on the differences in QoL between countries have focussed on validation of questionnaires across different countries rather than the actual difference in perceptions of QoL in different countries for a given disease [2-6]. There has been no direct comparison of QoL issues for MD in different countries. In performing our primary process of linguistic and cultural validation of INQoL for use in the United States we gained a unique opportunity to contrast and compare the UK and US experiences of those with MD and take this opportunity to present these results.

Although the use of validated QoL scales in MD does allow the collection of quantitative data that can be used in clinical studies and therapeutic trials, the process of reducing QoL to simple figures may obscure the experience of how living with MD really affects people. Qualitative research such as that required for the construction of questionnaires like INQoL does allow a closer appreciation of individuals' experiences of living with chronic disease and disability [7-9]. We therefore take this opportunity to report the verbal and written comments from both UK and US patients that provide unique insights into the actual impact of MD on their lives. While the UK quotes were the subject of qualitative analysis for the construction of the original INQoL, they have not been previously reported verbatim.

## Methods

UK patients were recruited from the MD clinic of King's College Hospital and from two UK muscle patient support groups. US patients were recruited from the muscle clinics of three US centres. Patients were eligible to take part in the study if their MD had been symptomatic for at least six months. MD diagnoses were confirmed through expert opinion using standard diagnostic criteria and confirmatory testing, including where appropriate molecular genetic analysis, serum creatine kinase levels, clinical neurophysiology studies, or muscle biopsy. Patients had to be aged 16 years and above and literate in English. Patients with major co-morbidity from other active cardiac, respiratory or rheumatologic disorders which would affect quality of life were excluded.

Patients were sent a questionnaire designed around the life domains that had been identified during the original UK semi-structured interviews [1]. For each domain there were closed ended questions asking the extent to which their MD affected this domain (on a five point Likert scale from 'not at all' affected to 'very much' affected) and the importance that they attached to this impact (on a five point Likert scale, ranging from 'not at all important' to 'extremely important'). We also asked whether the impact was "good" or "bad" and encouraged free text comments on the impact of MD on these life domains to allow respondents to provide greater detail of their experiences and include information not tapped by the close ended questions. The UK version of this questionnaire was independently converted into US English by the principal investigators from each of the three US centres (SP, JTK, CJ). Each US investigator then returned their version to the UK investigators (MR, VB) who collated the different versions, and resolved any inconsistencies by group discussion between all US and UK investigators to reach a consensus. Examples of divergence from the UK version included word changes such as; "tick" changed to "check", "stick" changed to "cane" and "colleague" changed to "co-worker".

We also collected data for respondents' gender, age and muscle disease diagnosis. The questionnaires were sent with a prepaid envelope for return. Non-responders were sent another questionnaire two weeks later and further non-responders were sent a reminder letter one week later. The study had Institutional Review Board approval from all the institutions involved.

### Analysis

For each life domain we compared the frequency distribution of UK versus US responses for both the extent of the impact and the importance of the impact. We also computed an overall impact score by assigning a ranking order to the product of the two factors (extent and importance) giving an overall impact scale from 1 to 9. Due to relatively large sample size and normal distribution we anticipated there would not be any difference between results of parametric and corresponding non-parametric tests; this was in fact the case. Thus parametric unpaired t-test analysis was performed to compare UK and US responses. Chi squared test was used to compare the UK and US responses for the direction of the impact, as good or bad. We performed Rasch analysis to examine the psychometric properties of the questionnaire in US and UK populations. In this model, responses to each question have a probabilistic correlation with the difficulty of the question [10]. We calculated fit of the observed data to the Rasch model and compared responses to questions in UK and US populations. In the course of the original UK construction of INQoL, interviews with 41 subjects were tape recorded and transcribed [1]. A phenomenological approach was used to guide the analysis and facilitate the exploration of patients' perceptions and experiences [8,11]. Themes were extracted and clustered together into categories representing life domains influenced by MD. A coding scheme was devised to represent the individual domain and sub-domain categories and this was applied to the data. Finally the validity of the coding scheme was verified through an external inspection by a second categorizer (AJC) who applied the scheme to a sample of interviews. We applied the same coding scheme to the free text comments given to us by both the UK and US respondents of the postal questionnaire. SPSS for Windows version 15.0 was used for statistical tests and Winstep version 3.68.2 was used to do the Rasch analysis.

## Results

The UK survey received 251 responses (response rate 47%) comprising 90 males and 161 females (ratio 1:2.8) ages 16- 96 (mean age 52.61, SD = 15.95). The US survey received 333 responses of which 10 had to be excluded because of missing data (effective response rate 50%). This sample comprised 176 males and 147 females (ratio 1.19:1) ages 18- 84 (mean age 50.15 years SD = 16.16) (Table 1). Diseases represented in both UK and US samples included congenital myopathies, limb girdle muscular dystrophies, facioscapulohumeral muscular dystrophy, dystrophic and non-dystrophic myotonias and inflammatory myopathies.

**Table 1:** Patient Demographics

Country	Gender	n	Age Average (SD)
UK	Male	64	55.61 (16.13)
	Female	182	51.48 (15.77)
	Total	249	52.62 (15.95)

US	Male	172	48.01 (16.69)
	Female	146	52.29 (15.41)
	Total	328	50.15 (16.16)

Total		577	51.22 (16.12)

SD: standard deviation

### Quantitative data

**Figure 1:**
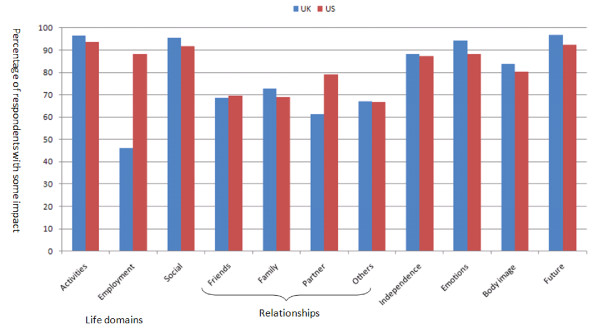
**The percentage of UK and US respondents endorsing some impact of their muscle disease on each of the life domains**.

The percentage of UK and US patients scoring some impact (i.e. scoring 2 to 5 for extent of impact), or no impact (scoring 1) for each of the domains is given in Figure 1. For the UK population the percentage reporting some impact ranged from 45% for Work to 96% for both Activities of Daily Living (ADL) and the Social and Leisure domain. For the US population the percentage reporting some impact ranged from 65% for Relationship with others to 94% for ADL.

**Figure 2:**
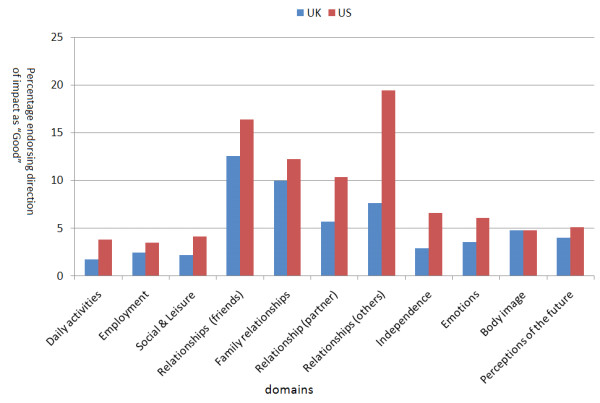
**The percentage of the UK and the US samples endorsing the direction of impact as being "Good" for each of the domains**.

Table 2 gives the impact scores (extent of impact, importance of impact and overall impact) for each of the life domains and the results of the significance tests between the UK and US samples. The overall importance score of all the relationship domains (Friends, Family, Others and Partners) were significantly higher in US patients compared to UK patients. This difference was not due to any difference in the extent of the impact but was due to there being a greater importance attached to the impact of MD on relationships by the US patients. US respondents scored a significantly higher impact in the Employment domain. For all domains the direction of the impact was negative for the majority of both UK and US respondents. What was interesting was that there was a significant minority who reported a positive impact of their muscle disease on relationships. (Figure 2).

**Table 2:** Descriptive data for domain scores from United Kingdom (UK) and United States of America (US)

		Extent		Importance		Overall	
		Mean	SD	Mean	SD	Mean	SD
Daily activities	UK	3.84	1.10	3.88	1.14	6.74	2.10
	US	3.65	1.17	3.92	1.00	6.76	1.73
	
Employment	UK	2.69*	1.76	2.68*	1.77	4.36*	3.48
	US	3.78	1.38	4.20	0.92	7.37	1.70
	
Social activities	UK	3.96*	1.13	3.80	1.17	6.76	2.18
	US	3.67	1.23	3.91	0.99	6.78	1.83
	
Relationships (friends)	UK	2.56	1.36	2.71*	1.48	4.27*	2.72
	US	2.60	1.35	3.35	1.22	5.43	2.15
	
Relationships (family)	UK	2.82	1.49	3.03*	1.59	4.84*	2.94
	US	2.71	1.44	3.73	1.13	6.04	2.07
	
Relationships (partner)	UK	2.66*	1.62	2.92*	1.76	4.56*	3.28
	US	3.15	1.48	4.05	1.08	6.70	1.98
	
Relationships (others)	UK	2.60	1.44	2.63*	1.46	4.23*	2.81
	US	2.45	1.33	3.33	1.17	5.26	2.14
	
Independence	UK	3.78*	1.42	4.05	1.34	6.81	2.65
	US	3.54	1.39	4.13	1.06	7.00	1.95
	
Emotions	UK	3.73	1.27	3.77	1.27	6.50	2.41
	US	3.38	1.36	3.84	1.09	6.50	2.07
	
Body image	UK	3.36	1.46	3.21	1.41	5.55	2.76
	US	3.06	1.45	3.47	1.21	6.00	2.29
	
Perceptions of the future	UK	4.13	1.07	4.07	1.13	7.19	2.06
	US	3.92	1.26	4.15	1.02	7.28	1.91

Extent column: Extent of Impact (minimum 1 and maximum 5); Importance column: Importance of impact (minimum 1 and maximum 5); Overall column: Overall impact (minimum 1 and maximum 9)

* significantly different (P < 0.01) (unpaired t- test)

SD: Standard deviation

Since the UK and US populations differed in gender distribution we performed unpaired t-test to explore the effects of gender on the responses. For all domains except "Body Image" males had higher impact scores compared to females. Females reported a significantly higher impact of their MD on "Body Image" domain than did males (Overall Impact score: mean female rank 260.7 versus male rank 218.48; P = 0.001). When this was separated out to look at UK and UK samples, UK female patients had higher impact on body image domain than UK male patients (P = 0.001); however there was no significant difference between US female and male patients. There was also no significant difference between UK and US female patients in body image impact score; but US male patients had a higher impact on body image than UK male patients (P = 0.005). The difference between body image scores in two populations is reduced by mixing UK male and female patients. Performing ANCOVA test considering gender as a covariate enabled us to control for the gender ratio difference in the two populations, and showed that the higher impact on employment and relationships in the US population remained and was therefore independent of the gender ratio difference: Employment (F = 25.925, P < 0.001), Relationships with friends (F = 14.865, P < 0.001), Family relationships (F = 12.529, P < 0.001), Relationship (partner) (F = 22.127, P < 0.001), Relationships with others (F = 10.312, P < 0.001), Body image (F = 8.926, P < 0.001), Perceptions of the future (F = 7.091, P = 0.001)).

We also explored the possible impact of age on impact scores by splitting the sample into those under and over 50 years-old. Those over 50 years-old had higher scores for all domains with the exception of the domain "Employment" for which those under 50 years-old scored a higher impact (Overall Impact score: mean rank for younger patients 220.46 and older patients 168.96; P value < 0.001).

### Rasch analsys

**Figure 3:**
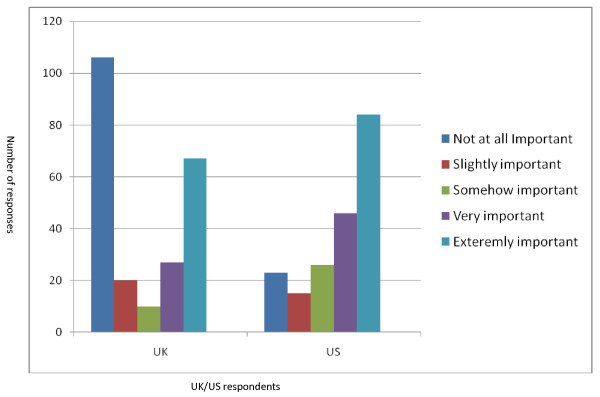
**Showing how UK and US sample differed in their responses for question 2C; "How important is the effect of your muscle disease on employment"**.

Rasch analysis gives outfit mean square values (msnq). Outfit msnq values represent the proportion of items "out of range" ie for which there is a higher proportion of "easy" or "difficult" responses than expected. Mean squares values between 0.70 and 1.50 are accepted as reasonably fit in psychological studies. On the whole, the questionnaire had very good outfit msnq (1.12). However the questionnaire items relating to "Work importance" and "Work impact" had outfit msnq values of 2.36 and 2.06 respectively meaning that they were the most "difficult" items for participants to endorse. This was mainly due to the effect of UK responses to the employment question as 106 patients (42% of UK population) answered "Employment importance" and "Employment impact" as "not important at all" as shown in Figure 3.

### Qualitative data

Table 3 summarises the specific issues raised by these patients and how they were clustered into the domains and sub-domains used for INQoL. The US respondents' free text comments gave them an opportunity to raise specific issues outside of the closed ended questions. In general these free text comments reflected issues already raised by UK patients and therefore already encompassed by the existing INQoL domains. Where additional specific issues were raised by US respondents, these also fitted into the existing domains. The quotes of patients illustrate the issues explored by INQoL, and provide insight into the real impact that MD has on these patients. In some cases the quotes from UK and US patients are remarkably similar. All the UK quotes including those in the sample given here were the ones grouped into the broad domains that made up the final INQoL UK questionnaire. These domains clearly corresponded across the UK and US surveys.

**Table 3:** The specific issues raised by the UK & US patients with muscle disease showing how these have been grouped into sub-domains and domains used by INQoL

Domain	Sub domain	Specific Issues raised by patients
Activities	Daily activities	Getting out and about (transport & driving)
		Self care
		Housework
		Falling
		
	Leisure	Sport
		Eating out
		Sedentary hobbies
		Visiting friends & family
		***Gardening***
		***Shopping***
		***Holidays/vacation***
		
	Employment	Work activities
		Stopping work
		Limitations to career/job prospects
		Discrimination
		Change in job/occupation
		Financial considerations
		
Social impact	Partner	Meeting potential partner
		Strain on relationships
		Sex life
		Support from partner (positive) ***Limits on doing activities with partner***
		
	Family	Lack of support
		Worry about being a burden
		Worry about passing on gene
		Support from family (positive) ***Lack of understanding*** ***Limits on participation***
		
		
	Friends	Difficulty in visiting friends
		Loss of contact
		Support from friends (positive)
		
	General social interaction	Avoidance of situation
		Lack of understanding
		Difficulty explaining condition
		Other people's perceptions
		Problems of access
		Discrimination
		Encouragement from others (positive) ***Hidden disease*** ***Other people's emotions*** ***Difficult to plan (unpredictable)***
		
Psychological Impact	Emotions	Feelings of loss
		Feelings of abandonment
		Guilt
		- at not being able to fulfil social role
		- at passing on gene to children
		Depression
		Frustration
		Sadness
		***Anxiety***
		***Fear***
		***Shame/embarrassment/self-conscious***
		***Loneliness***
		
	Perception of the future	Fear of losing independence
		Fear of passing on gene to children (congenital patients)
		***Awareness that future is uncertain***
		
	Identity/Self-image	Body image
		Social image/identity/role
	Independence	Loss of control
	Coping strategies	Determination
		Acceptance
		- of condition
		- that no treatment available
		Focusing on the present
		Making the best
		Taking control
		Changing expectations
		Planning
		Religious faith
		Social comparison
		Avoidance of situations/people/places
		Covering effects of condition
		Attempts to carry on as before
		***Avoidance of specific entities that are believed to worsen condition (stress, people with infections, pollen, extreme temperatures)***
		***Hope for the future***
		***Pacing self/resting to adapt to tiredness/adjust to accommodate***

The specific issues raised by the US population given in bold italics are different to those raised by UK subject but fit within the existing sub-domains and domains as shown.

#### Daily activities

Remarks about daily activities ranged from those about personal care to others about housework, getting around and shopping. To some extent the functional effects of muscle weakness on daily activities were predictable and easily related to the likely distribution of weakness in these patients. This quote

"*I do have problems still when blow-drying my hair. When I hold the hairdryer for a long period, my arms start to feel heavy and tired." - UK patient*

reflects proximal upper limb weakness while this one

"*Difficulty in getting from a sitting position to a standing position, difficulty in retrieving an object off the floor, difficulty in use of fingers to grip objects, to write a letter, to do personal hygiene, difficulty walking without aid and decreased stamina when ambulating for long periods of time (20-30 min)." - US patient*

reflects mostly proximal lower limb weakness but with some hand weakness also. What may be less appreciated by medical professionals is the degree to which fatigue and pain impact upon daily activities as evidenced by two similar quotes from UK and US patients;

"I do not have the strength/energy to sustain normal day-to-day living activities for more than a couple of hours. I become overwhelmingly tired and my muscles ache so much I cannot do anything or even concentrate on sedentary activities. This is despite painkillers. I only recover after lying down for a couple of hours. It takes so much effort to get through the basic needs of living that there is no energy/time left for social activities. The almost constant pain affects concentration too." - UK patient

"I am not able to participate in sports anymore and at social functions, around 2-3 pm, I fall asleep." - US patient

#### Employment

Many comments were made about respondents' working life. These related not only to the effects of MD upon working activities but also upon job prospects;

"I was made redundant three years ago when the company I had worked with for 17 years relocated. I firmly believe that the reason I was not able to get another permanent job was because of my condition." - UK patient

"I lost my job due to weakness & fatigue. Most of my co-workers were working 12 hour shifts. There is no way I could do that. I loved my job & did not want to go on disability (benefits)."-UK patient

Many respondents commented on their having to take early retirement on ill health grounds, or upon the adaptations they had made to their jobs or career path. The impact of stopping work upon self-identity was clear;

"(I) Had to retire from work at age 35 - (this) had a huge impact on how I saw myself as a contributing member of society." - UK patient

"(I) Had to retire at 53 because of my muscle problems - loss of 60% of my income & my health insurance was cancelled after 30 years at the same company." - US patient

#### Social and leisure activities

Respondents also commented upon the impact of their condition upon leisure and social activities. Both sporting and sedentary activities were influenced. Difficulties socialising and visiting friends and family were commonly reported and these were linked to transport problems and difficulties in accessing buildings and homes;

"This illness affects all my leisure activities, as I'm in a wheelchair. I've had to give up dancing, gardening and even visiting friends and shopping. Even my son and daughter I cannot visit as there are steps which I cannot get up over. Some shops have a ramp or are level but a lot aren't. I have been confined to the house for 5 months due to the fact I could no longer transfer from the car to my wheelchair. After 5 months of worry and expense we have just had a car converted for me to drive." - UK patient

"It's hard to do some of the things I like to do, like go for walks, ride bike and hang out with my friends." US patient

#### Relationships and social interactions

Some respondents commented that friendships had faded as a result of the difficulties in taking part in activities previously shared with friends;

*"A lot of "friends" simply drift away when it is obvious there is a chronic condition which is unlikely to change, and when one can no longer go out and about to mutual interests or be relied on to entertain at home. Someone who would rather go to bed than even sit and talk becomes of little interest to any but the most long suffering of friends." *UK patient.

Many respondents felt that there was a lack of understanding about MD and its effects. This made the condition difficult to explain. In particular patients with myositis frequently commented upon the difficulty they had in explaining their condition to other people. This was believed to be due to their appearance of physical well-being;

"The main problems lie with the condition not showing any visible signs and symptoms and often having to explain oneself or making excuses for inabilities." - UK patient

"Many people do not see my handicap on the surface. My job does not entail heavy lifting, getting up and down heavy stairs or ladders, so many do not know of my handicap. Only when we are invited to participate in activities where there will be physical activity will I make excuses not to." - US patient

Some patients also commented upon the lack of understanding about their condition, not only in other people but also in the in medical profession. This was a source of great distress;

"They and also medical staff have never heard of "Inclusion Body Myositis" - UK patient

*"You're not that bad." - US patient referring to comments about relationships & social interaction*.

*"Most people don't understand Inclusion Body Myositis. Of course, this is also true of doctors" - US patient*.

#### Family

An immediate effect upon some respondents' behaviour towards their family was evident;

"I find myself distancing myself from my family. If I am too caring I may be expected to physically do something to show that I love them. I used to do it in the past, even though I suffered for it- their needs were met whilst I cried alone in pain. I'm continually trying to balance my relationships but this year have felt, I just want to be left alone so that I can at least cope and have enough energy to be reasonably cheerful and good company for my husband who I am sure you appreciate has had a lot to cope with himself. To be honest I don't answer the phone or the door while my husband is at work and when he comes home I let him do it." - UK patient

*"Due to my limits I find I am a bit more concerned about my relationships with other close family members. I require more from them." - US patient*.

Problems of access to the homes of friends and family and to social venues were also a source of difficulty for relationships.

#### Partners

A large proportion of the respondents commented upon the impact their condition had exerted on their relationship with their spouse or partner;

*"My muscle condition, weakness (and) tiredness destroyed my marriage in the end. I'm now on my own and can see no future with myself ever having a relationship with anyone ever again, it's just too hard." - UK patient*.

"My spouse divorced me. It's been about two months. We were married for 24 years." - US patient

"We don't share activities any more, we can't go on same kind of holidays we used to, I'm a nuisance when we go out together, and I've gone off sex completely. I can't see why he still bothers with me." - UK patient

*"How could it not affect spouse. Walking slowly, vacation planning, lack of spousal relationships, loss of 60% income - wife had to go back to work & my only son just started college." - US patient*.

A number of respondents also commented upon the difficulties they had in meeting potential partners;

"When it comes to a partner, everything is fine until I mention my illness, in which case they don't want to know." - UK patient

"I am filing for a not to be with my wife & I am having a hard time with getting or staying with someone because of this." - US patient

However, the support provided by respondents' families and partners was also clear from a number of comments;

"When I finally get diagnosed my fiancé and friends were really supportive and helped me to get through the most difficult times." - UK patient

"My family & friends are very supportive of my MD in that they help with stairs, getting up from chairs etc." - US patient

The themes relating to emotions and other psychological issues were very much intertwined and respondents tended to comment upon the emotional impact of MD in the context of the other life domains. For example, patients' fears about the future had a considerable impact upon the emotional feelings expressed.

#### Independence

Independence was alluded to a great deal by both UK and US subjects;

"I have to totally depend on others for just about everything. I can use my hands to eat and drive my mobile chair. I can't comb my own hair; I need help to take a bath, use the toilet and to get in and out of bed. Also to sit up. I am totally dependent on others. I can take my own medicine whilst sitting." - US patient

Independence was sometimes mentioned with particular reference to one's spouse;

"I am very dependent on my husband and this affects me emotionally. I feel a burden sometimes, although he doesn't complain." - UK patient

Independence was also intertwined with perceptions about appearance, fears about the future and religiosity as coping strategy;.

"My independence is gone, that is what hurts most of all. I used to be proud of the way I used to look, but not now with my weight loss. The little use of my hands, feet, throat and neck control, it's hard to think of what the future might bring. I just ask my Lord to walk with me and help me carry on"... - US patient

"Sometimes I feel well balanced and happy and yet other times I feel sad and lonely even though I have a wonderful family. I picture my future as being on my own, ill and more dependent than I am now, but I try not to think about the future." - UK patient

#### Perceptions of the future

Patient's perceptions of the future were linked to becoming a burden on family;

"My thoughts about the future are very fearful. I know this illness is supposed to be slow in progressing, but how slow? At the moment I can only stand for a few seconds and if I dare bend my knees I'm on the floor in one untidy heap and can only get up with my husband's help (and) with a hoist. So I cannot walk or stand. I cannot lift my head off the pillow when I'm in bed and it's very difficult to turn over in bed... what happens if I get worse. My husband is seventy-five. What will happen if he dies?" - UK patient

"What future independence! I'll be getting weaker and end up in a wheelchair with people waiting on me." - US patient

Other concerns about the future related to the genetic nature of some muscle diseases. A number of respondents were worried about the possibility that they had passed on their condition to their children. They also had concerns about being able to physically care for their children in the future;

"I tend to worry a lot about how much of a burden I will become to my family, my wife especially, as I get more immobile. Also I worry a great deal about what I have passed onto my daughter. They learnt about genetics at school, so they know that it is passed on, but I was told by a doctor at an FSH (facioscapulohumeral muscular dystrophy) get together not to have them tested unless it was absolutely necessary as the tests were not always conclusive." - UK patient

"I have a little boy, who I feel may have my condition or worse. I can't imagine how hard it would be for me to take care of him if my condition should happen to worsen. That is my everyday worry - towards the future." - US patient

#### Self Image, identity and body image

MD itself, but also its treatment, had effects on actual physical appearance;

"I hate the way long-term steroid medication has made me look. I cannot bear to look in a mirror and I just cannot look at photos of myself taken before my condition." - UK patient

In other cases the effect on body image was more to do with the way it affected one's mobility;

"I feel like a second class citizen as I use a walker and I feel like people are looking down on me because I have a problem walking and getting around." - US patient

Loss of independence and its consequent dependence on others had a considerable impact upon patients' self-image and sense of identity;

"I don't really have any independence. I think I must have lost the will to even want to be independent. I don't feel like I'm a person any more- maybe just an extension of my husband most of the time, which is not so bad because he's a really nice guy!" - UK patient

Subjects' perceived poor body image could also contribute to social isolation;

"(I) Stay away from people that know about my condition, then I am just me." - US patient

#### Sex

The impact of MD upon body image also had implications for social and sexual confidence;

"I'm very self-conscious when it comes to the sex side of the relationship and I want to hide myself as much as possible. Consequently I find myself "putting off" the physical side of the relationship and so not encourage it even though we both still enjoy it when it does happen. I'm a bit overweight because of lack of exercise so I am trying to cut down my intake of food." - UK patient

Physical impairments also made sexual activity difficult for some;

"The only deleterious effect on my relationships has to do with my wife. It prevents me from being as agile as I would prefer during sex." UK patient

#### Coping

A variety of recognised coping strategies were employed by those with MD. These included a) planning;

"Because of the condition it is not easy to do things on the spur of the moment. It is not always practical to go to places that are new to me without a bit of forward planning. For example, are there stairs or lifts, how far is it to walk, what is the ground like?" - UK patient

"...when I fly I order a town car to take me to the airport, I order a wheelchair to be waiting at the church and order another for my destination... I get into a car better on the driver's side, so I request that I sit behind the driver..." - US patient

b) focusing on the immediate day to day issues with a element of denial of the future;

"The future frightens me, so I have trained myself not to think too far ahead and to enjoy what independence I have today." - UK patient

"I live day by day and am doing fine." UK patient

c) comparing one's self with less fortunate individuals;

"However, I consider myself blessed and try to use my heart to be of help to others in worse shape than I." - US patient

"After reading what other folk have gone through I feel very fortunate to be as healthy as I am." - UK patient

d) remaining optimistic;

"I am optimistic about the future, which will involve study, more sedentary hobbies and refusing to be beaten by this disease." - UK patient

"Well I am a complete optimist and I go day by day so I feel rather good on emotion since I don't get as much pain as I used to and I can handle the tremor. I am doing all right." - US patient

e) acceptance and changing expectations;

"One has to accept that there are many things one can no longer do- if these are very important one has to ask for help- if not the there is a conscious decision to do without or accept a lowering of expectations and a compromise on previously acceptable standards." - UK patient

f) religion;

"At the age of 72, my best way to maintain peace/comfort is with my good wife and prayer." UK patient

## Discussion

The results of this analysis show that the quality of life domains used by INQoL effectively captures the experiences of both UK and US patients with MD. The quantitative data shows that all the domains were embraced by US patients as they were by UK patients. The qualitative data suggests that the issues raised by both UK and US populations are very similar and no new items were identified by the US survey that could not reasonably be fitted into the domains already chosen. As with all such studies selection and responder biases raise questions about the generalisability of our findings but the sample sizes are large for these rare diseases and the findings are in accordance with our clinical experience. For the majority of both UK and US patients muscle disease had a detrimental effect on quality of life domains but a significant minority reported a beneficial effect on relationships. In interviews this minority reported that their muscle disease had made them closer to family and friends for a variety of reasons.

The significant quantitative differences in the domain impact responses between the two countries are of interest but cannot be assumed to be simply due to cross cultural differences. There are a number of potential differences between the UK and US populations that may have accounted for different response rates. One known difference between the UK and US populations is gender distribution. Our analysis suggests that this may explain some of the differences in that males scored a higher impact for most domains than did females with the exception of "Body Image" where females scored higher. The two samples were fairly similar in age distribution and so the differences in responses we found between those under and over 50 years-old seem unlikely to account for any difference in responses of the UK versus US populations' responses. Similar issues were raised in a study of the effect of epilepsy on psycho-social factors which showed US, New Zealand and UK subjects had some different scores but that interpretation of these was confounded by various demographic variables and the unknown differences in the type and severity of the epilepsy in the three national groups [12].

Other important factors to consider would be the distribution of specific muscle disease diagnoses, and the range of muscle disease severity. The UK and US population were recruited in different ways which is likely to have led to differences in disease distribution and severity. Such differences may have had an important bearing upon interpretation of any differences in impact scores not just those between countries but also those between other groups such as gender. That females report more impact of their MD on Body Image might be considered not surprising. However, in order to fully appreciate whether this is a true gender difference, one would have to take account of the specific muscle diseases present in the male and female populations and the severity of such diseases. Some muscle diseases have more obvious effect on Body Image than others. Facial features such as facial weakness in facioscapulohumeral dystrophy, or ptosis in myotonic dystrophy may mean these diseases impact more on Body Image than does limb girdle muscular dystrophy which usually has no facial involvement. Inflammatory muscle diseases are treated with steroids which may have side effects such as weight gain, moon face, hirsuitism, and buffalo hump that may disproportionately affect Body Image. More severe muscle disease affects the gait and movements generally and can of course mean that one is obliged to use a wheelchair. Some patients referred to these aspects when explaining the impact of their MD on Body Image.

If however the differences in disease impact recorded by UK and US populations reflect true cross cultural differences, this raises further interesting questions for research. The greater importance that the US population attaches to Relations with family and friends may reflect differing societal values. The lesser impact of muscle disease on Employment in the UK may be due to the fact that unemployed UK patients are better supported by the social security system such that lack of employment has less impact and importance for them. Conversely we had expected some differences between the UK and the US that did not in fact materialise. The impact of muscle disease on Activities was the same for UK and US despite the longer duration of disability access legislation in the US and the higher proportion of spacious urban living in the US as compared with the higher proportion of crowded city living in the UK. The influence of differences in health care systems, social care, support networks and cultural perceptions on QoL in different countries could be the subject of further illuminating research. Lessons from different countries might then shape public and social policy for the benefit of those with MD.

A practical challenge that arises from any future MD research in this field concerns the heterogeneity of MD. Collectively MD is common but it is made up of a variety of individual diseases each of which may each be rare. It is reasonable to study them collectively as all MD may have common symptoms of weakness, fatigue, or pain and doing so means one can obtain sufficient numbers for quantitative research. However the individual MDs do differ in their age of onset, distribution of weakness, speed of progression and severity of symptoms. For some questions controlling for all these factors in future research may be challenging. Only future research will tell us to what degree lumping or splitting of the muscle diseases is required for quality of life research.

## Conclusions

INQoL is appropriate for use in US population but there may be differences in the importance that US subject attach to certain aspects of QoL that could be the basis for further study.

If these differences are confirmed then this may have implications for the interpretation of QoL outcomes in multi-national trials.

## Abbreviations used

ADL: Activities of Daily Living; INQoL Individualised Neuromuscular QoL; QoL: Quality of Life; MD: Muscle disease; UK: United Kingdom; US: United States of America.

## Completing interests

The authors declare that they have no competing interests.

## Authors' contributions

RS helped draft the paper and did the statistical analysis. KAV, AJC and JW collected and analysed the UK data. SP, JTK and CJ collected the US data. VB collated and performed qualitative analysis on the US data. MRR was principle grant holder for the project and drafted the paper. All authors are members of the Muscle Study Group that helped initiate this research. All authors read and approved the final manuscript.
